# A partially randomised trial of pretomanid, moxifloxacin and pyrazinamide for pulmonary TB

**DOI:** 10.5588/ijtld.20.0513

**Published:** 2021-04-01

**Authors:** C. D. Tweed, G. H. Wills, A. M. Crook, E. Amukoye, V. Balanag, A. Y.L. Ban, A. L.C. Bateson, M. C. Betteridge, W. Brumskine, J. Caoili, R. E. Chaisson, M. Cevik, F. Conradie, R. Dawson, A. del Parigi, A. Diacon, D. E. Everitt, S.M. Fabiane, R. Hunt, A. I. Ismail, U. Lalloo, L. Lombard, C. Louw, M. Malahleha, T. D. McHugh, C. M. Mendel, F. Mhimbira, R. N. Moodliar, V. Nduba, A. J. Nunn, I. Sabi, M. A. Sebe, R. A. P. Selepe, S. Staples, S. Swindells, C. H. van Niekerk, E. Variava, M. Spigelman, S. H. Gillespie

**Affiliations:** 1 Medical Research Council Clinical Trials Unit, University College London (UCL), London, UK; 2 Centre for Respiratory Disease Research, Kenya Medical Research Institute (KEMRI), Kenyatta National Hospital, Nairobi, Kenya; 3 Lung Center of the Philippines, National Centre for Pulmonary Research, Quezon City, The Philippines; 4 Pusat Perubatan Universiti Kebangsaan, Kuala Lumpur, Malaysia; 5 Centre for Clinical Microbiology, UCL, London, UK; 6 Global Alliance for TB Drug Development, New York, NY, USA; 7 Aurum Institute, Rustenburg, South Africa; 8 Tropical Disease Foundation, Makati Medical Centre, Makati City, Phillippines; 9 School of Medicine, Johns Hopkins University, Baltimore, MD, USA; 10 Medical School, University of St Andrews, St Andrews, UK; 11 University of the Witwatersrand, Clinical HIV Research Unit, Johannesburg, South Africa; 12 University of Cape Town Lung Institute, Cape Town, South Africa; 13 TASK Applied Science, Bellville, South Africa & Division of Physiology, Department of Medical Biochemistry, University of Stellenbosch, Tygerberg, South Africa; 14 Universiti Teknologi MARA, Selangor, Malaysia; 15 Enhancing Care Foundation, Durban International Clinical Research Site, Wentworth Hospital, Durban, South Africa; 16 Madibeng Centre for Research, Brits, & Department of Family Medicine, University of Pretoria, Pretoria, South Africa; 17 Setshaba Research Centre, Soshanguve, South Africa; 18 Ifakara Health Institute (IHI), Dar es Salaam, Tanzania; 19 THINK (Tuberculosis and HIV Investigative Network), Durban, South Africa; 20 KEMRI, Nairobi, Kenya; 21 Mbeya Medical Research Center, National Institute for Medical Research, Mbeya, Tanzania; 22 The Aurum Institute, Tembisa Clinical Research Centre, Tembisa, South Africa; 23 The Aurum Institute, Klerksdorp, South Africa; 24 Department of Internal Medicine, University of Nebraska Medical Center, Omaha, NE, USA; 25 Global Alliance for TB Drug Development, Pretoria, South Africa; 26 Klerksdorp Tshepong Hospital, Klerksdorp, South Africa

**Keywords:** tuberculosis, drug resistance, TB treatment, TB-HIV

## Abstract

**BACKGROUND:**

Treatment for TB is lengthy and toxic, and new regimens are needed.

**METHODS:**

Participants with pulmonary drug-susceptible TB (DS-TB) were randomised to receive: 200 mg pretomanid (Pa, PMD) daily, 400 mg moxifloxacin (M) and 1500 mg pyrazinamide (Z) for 6 months (6Pa_200_MZ) or 4 months (4Pa_200_MZ); 100 mg pretomanid daily for 4 months in the same combination (4Pa_100_MZ); or standard DS-TB treatment for 6 months. The primary outcome was treatment failure or relapse at 12 months post-randomisation. The non-inferiority margin for between-group differences was 12.0%. Recruitment was paused following three deaths and not resumed.

**RESULTS:**

Respectively 4/47 (8.5%), 11/57 (19.3%), 14/52 (26.9%) and 1/53 (1.9%) DS-TB outcomes were unfavourable in patients on 6Pa_200_MZ, 4Pa_200_MZ, 4Pa_100_MZ and controls. There was a 6.6% (95% CI −2.2% to 15.4%) difference per protocol and 9.9% (95%CI −4.1% to 23.9%) modified intention-to-treat difference in unfavourable responses between the control and 6Pa_200_MZ arms. Grade 3+ adverse events affected 68/203 (33.5%) receiving experimental regimens, and 19/68 (27.9%) on control. Ten of 203 (4.9%) participants on experimental arms and 2/68 (2.9%) controls died.

**CONCLUSION:**

PaMZ regimens did not achieve non-inferiority in this under-powered trial. An ongoing evaluation of PMD remains a priority.

## Abstract (FR)

**CONTEXTE:**

Le traitement de la TB est long et toxique et de nouveaux protocoles sont requis.

**MÉTHODES:**

Des patients atteints de TB pulmonaire sensible aux médicaments (DS-TB) ont été randomisés pour recevoir : 200 mg de prétomanide (Pa, PMD) quotidien, 400 mg de moxifloxacine (M) et 1500 mg de pyrazinamide (Z) pendant 6 mois (6Pa_200_MZ) ou pendant 4 mois (4Pa_200_MZ) ; 100 mg de PMD quotidien pendant 4 mois dans la même combinaison (4Pa_100_MZ) ; ou un traitement standard de DS-TB pendant 6 mois. Le résultat principal a été l’échec du traitement ou la rechute à 12 mois après la randomisation. La marge de non infériorité pour les différences entre groupes a été de 12,0%. Le recrutement a été mis en pause à la suite de trois décès et n’a pas été repris.

**RÉSULTATS:**

Quatre sur 47 (8,5%), 11 sur 57 (19,3%), 14 sur 52 (26,9%) et 1 sur 53 (1,9%) des résultats de DS-TB ont été défavorables pour 6Pa_200_MZ, 4Pa_200_MZ, 4Pa_100_MZ, et les témoins. Il y a eu une différence de 6,6% (IC 95% −2,2 à 15,4) par protocole et 9,9% (IC 95% −4,1 à 23,9) en réponse défavorable entre les témoins et 6Pa_200_MZ. Des effets secondaires de grade 3+ ont affecté 68 patients sur 203 (33,5%) recevant des protocoles expérimentaux et 19 sur 68 (27,9%) on control. Dix sur 203 (4,9%) participants en bras expérimental et 2 sur 68 (2,9%) on control sont décédés.

**CONCLUSION:**

Les protocoles Pa_200_MZ n’ont pas atteint la non infériorité dans cet essai sous-puissant. La priorité reste l’évaluation du PMD.

TB is the leading cause of death from an infectious disease globally.^1^ TB treatment for drug-susceptible and -resistant disease is hindered by its long duration and toxicity.^2^ Pretomanid (PMD, Pa) is a member of the nitroimadazole drug class^3^ that has demonstrated significant bactericidal and sterilising activity against *Mycobacterium tuberculosis*.^4,5^ PMD has recently been approved by the US Food and Drug Administration (FDA) as part of a 6-month regimen in combination with bedaquiline (BDQ) and linezolid for the treatment of extensively drug-resistant TB (XDR-TB) and treatment-intolerant or non-responsive drug-resistant TB (DR-TB).

PMD, moxifloxacin (MFX, M) and pyrazinamide (PZA, Z) were studied in a Phase 2 clinical trial in combination, with promising results for 8-week bactericidal activity.^6^ The STAND (Shortening Treatment by Advancing Novel Drugs) trial investigated the efficacy and safety of PaMZ for the treatment of both drug-susceptible (DS) and rifampicin-resistant (RR) pulmonary TB.

## METHODS

### Study design

The study was designed as a partially randomised, open-label, non-inferiority Phase 3 clinical trial comparing three experimental treatment regimens against standard TB treatment for pulmonary DS-TB. RR-TB cases were allocated to a separate treatment arm without randomisation (Clinicaltrials.gov number NCT02342886). There were 27 sites across South Africa, Tanzania, the Philippines, Kenya, Malaysia, Uganda, Thailand and Ukraine. The trial protocol, laboratory manual and statistical analysis plan are available at https://www.tballiance.org/portfolio/trial/5091. A full list of ethics committee approvals is included in Supplementary Data (Clinicaltrials.gov number NCT02342886).

### Study participants

Participants were adults (≥18 years), and had sputum 1+ or greater (International Union Against Tuberculosis and Lung Disease/WHO scale). Baseline alanine aminotransferase (ALT) or aspartate aminotransferase (AST) of ≥3 times the upper limit of normal (xULN), or a total bilirubin >2xULN (other liver tests normal) or >1.5xULN (other liver tests abnormal) were exclusion criteria. HIV-positive participants with CD4+ counts <100 cells/mm^3^ or WHO Stage 4 disease were excluded. The inclusion and exclusion criteria can be found in Section 5 of the Supplementary Data.

### Randomisation and study treatments

Participants with DS-TB were randomised to one of four treatment arms using online randomisation software in a 1:1:1:1 ratio: 200 mg PMD, 400 mg MFX and 1500 mg PZA daily for either 6 months (6Pa_200_MZ) or 4 months (4Pa_200_MZ); 100mg pretomanid for 4 months in the same combination daily (4Pa_100_MZ); or isoniazid (INH, H), rifampicin (RIF, R), PZA and ethambutol (EMB, E) daily for 8 weeks, followed by INH and RIF (HR) daily for 18 weeks (2HRZE/4HR) as detailed in the Supplementary Data Section 7.2. All participants with RR-TB were assigned to receive the 6Pa_200_MZ regimen.

The method of randomisation used for the DS-TB patients was minimisation with random element. Minimisation factors were centre, HIV status and presence or absence of cavities on local chest X-ray.

### Laboratory methods

Rapid molecular testing was used to assess INH, RIF, fluoroquinolone (FQ) and PZA susceptibility at screening (GenoType MTBDR*plus* [Hain Lifescience, Nehren, Germany], Xpert^®^ MTB/RIF [Cepheid, Sunnyvale, CA, USA], GenoType MTBDR*sl* [Hain Lifescience] and *pnc*A genotyping). Mycobacterial liquid culture was performed using MGIT^™^ (BD, Franklin Lakes, NJ, USA), and phenotypic drug susceptibility testing (DST) for streptomycin (SM), INH, RIF, EMB, PZA and MFX was confirmed using MGIT. The Laboratory Manual has been included in the Supplementary Data. DS-TB cases were susceptible to HRZ and FQs. Participants with RR-TB were confirmed as RIF-resistant, PZA-susceptible and FQ-susceptible, and either INH-susceptible or INH-resistant.

### Study procedures

Chest X-rays were performed during screening. At each study visit, sputum samples were obtained, a physical examination performed and information on adverse events (AEs) collected. Electrocardiograms were performed at pre-defined visits and at the site doctor’s discretion (see Section 8 in the Supplementary Data for the protocol visit schedule). AEs were graded according to the Division of Microbiology and Infectious Diseases (National Institutes of Health, Bethesda, MD, USA) criteria (available at https://www.niaid.nih.gov/sites/default/files/dmidadulttox.pdf) with an assessment of relationship to study drugs made by the investigator. AEs were considered “treatment-emergent” or “on-treatment” if they occurred in the period from first dose of trial drug up to 14 days after last dose. Follow-up was for 24 months after randomisation (see Section 13 in the Supplementary Data).

### Study outcomes

The primary efficacy, “unfavourable” outcome, was the proportion of participants with bacteriologically or clinically defined treatment failure or relapse 12 months after randomisation (from 50 to 54 weeks). A “favourable” outcome was defined as having a negative culture status (defined as two consecutive negative culture results at least 1 week apart with no intervening positive result) at 12 months if not already classified as having an unfavourable outcome. Details are provided in Table 1. While patients favourable or unfavourable under modified intention to treat (mITT) may be excluded from the per-protocol (PP) analysis, unfavourable patients cannot become favourable under PP and vice versa. Relapse was declared if positive cultures after the end of treatment were considered identical to the baseline sample by whole-genome sequencing (WGS) (<20 single nucleotide polymorphisms difference), or if WGS was not available. Recurrence was considered to be re-infection if the *Mycobacterium tuberculosis* strain was different by >100 SNPs from the baseline strain.

**Table 1:** Trial analysis populations and related unfavourable outcomes: list of definitions applied at end-point review for patients in the trial by analysis population *

Modified intention-to-treat population
All randomised patients included, except: Late exclusions due to resistance pattern, lack of culture confirmation, protocol violation at enrolment Patients who, having completed treatment, are lost to follow-up or withdrawn from the study with their last status being culture-negative Women who become pregnant during treatment and stop their allocated treatment Patients who died during treatment from violent or accidental cause Patients who died during follow-up (after the end of treatment) with no evidence of failure or relapse of their TB Patients who, after being classified as having culture-negative status, are re-infected with a strain other than that with which they had been originally infected Patients who are able to produce sputum at 12 months, but whose 12-month visit sputum samples are all contaminated or missing, who cannot be brought back for repeat culture testing^†^ Unfavourable outcome definitions Patients not classified as having achieved or maintained culture-negative status when last seen Patients previously classified as having culture-negative status who, following the end of treatment, have two positive cultures without an intervening negative culture Patients who had a positive culture not followed by at least two negative cultures when last seen Patients dying from any cause during the 6-month treatment phase, except from violent or accidental cause (e.g., road traffic accident), not including suicide (e.g., suicide was considered an unfavourable outcome) Patients definitely or possibly dying from TB-related cause during the follow-up phase Patients requiring a restart or a change of treatment because of an unfavourable outcome with or without bacteriological confirmation, i.e., on bacteriological, radiographic or clinical grounds Patients requiring an extension of their treatment beyond that permitted by the protocol, a restart or a change of treatment for any reason except reinfection or pregnancy Patients failing to complete an adequate course of treatment, who were unassessable at 12 months Patients lost to follow-up or withdrawn from the study before the end of treatment
Per protocol population

All randomised patients included, except: Late exclusions due to resistance pattern, lack of culture confirmation, protocol violation at enrolment Patients who, having completed treatment, are lost to follow-up or withdrawn from the study with their last status being culture-negative Women who become pregnant during treatment and stop their allocated treatment Patients who died during treatment from violent or accidental cause Patients who died during follow-up (after the end of treatment) with no evidence of treatment failure or relapse of their TB Patients who, after being classified as having culture-negative status, are re-infected with a strain other than that with which they had been originally infected Patients who are able to produce sputum at 12 months, but whose 12-month visit sputum samples are all contaminated or missing, who cannot be brought back for repeat cultures^†^ Patients lost to follow-up or withdrawn before the end of treatment^†^ Patients whose treatment was modified or extended for reasons other than an unfavourable therapeutic response to treatment^†^ Patients not meeting the definition of having received an adequate amount of their allocated study regimen (80% of treatment by self-reporting)^†^ Patients who are classified as “major protocol deviations”^†^ Unfavourable outcome definitions: Patients not classified as having achieved or maintained culture-negative status when last seen Patients previously classified as having culture-negative status who, following the end of treatment, have two positive cultures without an intervening negative culture Patients who had a positive culture not followed by at least two negative cultures when last seen Patients dying from any cause during the 6 month treatment phase, except from violent or accidental cause (e.g., road traffic accident), not including suicide (e.g., suicide will be considered an unfavourable outcome) Patients definitely or possibly dying from TB related cause during the follow-up phase Patients requiring a restart or a change of treatment because of an unfavourable outcome with or without bacteriological confirmation, i.e., on bacteriological, radiographic or clinical grounds

* Relapse was declared if positive cultures after the end of treatment were considered identical to the baseline sample using WGS (difference of <20 SNPs), or if WGS was not available. Recurrence was considered to be re-infection if the *M. tuberculosis* strain was different by >100 SNPs from the baseline strain.

^†^ Unless already declared as unfavourable.

WGS = whole-genome sequencing; SNP = single-nucleotide polymorphism.

Secondary efficacy endpoints included time to an unfavourable outcome, time to culture-negative status, and proportions of treatment failure or relapse 24 months after randomisation. The primary safety outcome was the proportion of participants with one or more Grade 3 or 4 AEs.

### Study oversight

An independent data safety monitoring committee (DSMC) of clinicians and statisticians reviewed unblinded data and oversaw the conduct of the trial.

### Statistical analysis and sample size

A sample size of 300 participants recruited to each DS-TB treatment arm was calculated to provide 90% power to show non-inferiority, with a margin of 12% in the upper boundary of the two-sided 95% Wald confidence interval (CI) for the proportion with unfavourable outcome and a one-sided significance level of 2.5%.

To preserve the overall type I error, a hierarchical approach to the analysis was adopted. The first comparison was 6Pa_200_MZ vs. the control arm. Comparison between the control and the 4-month arms would only be made if this was found to be non-inferior. Non-inferiority would only be demonstrated if indicated in both the mITT and PP analyses. Table 1 lists the definitions for mITT and PP populations in the trial. Participants who received at least one dose of trial medication were included in the safety analysis.

## RESULTS

### Study participants

**Figure 1:**
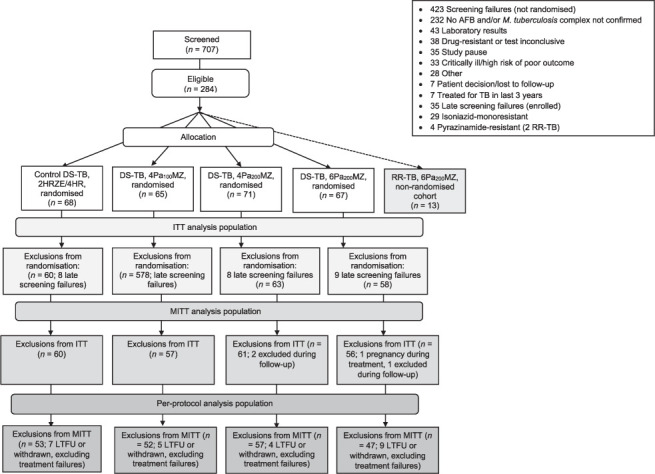
CONSORT diagram, indicating randomisations and exclusions. Patients with RR-TB were not randomised and allocated to receive 6MPa_200_Z. Pa 200 mg daily, M 400 mg and Z 1500 mg for 6 months (6Pa_200_MZ) or 4 months (4Pa_200_MZ); Pa 100 mg daily for 4 months in the same combination (4Pa_100_MZ). Late identification of drug resistance and withdrawal of consent were the most common reasons for exclusion during follow-up. AFB = acid-fast bacilli; RR-TB = rifampicin-resistant TB; DS-TB = drug-susceptible TB; H = isoniazid; R = rifampicin; Z = pyrazinamide; E = ethambutol; Pa = pretomanid; M = moxifloxacin; ITT= intention-to-treat; MITT = modified ITT; LTFU = lost to follow-up. CONSORT = Consolidated Standards of Reporting Trials.

The recruitment disposition of the participants in the trial is shown in Figure 1 with 271 of planned 1,200 patients randomised and 13 RR-TB patients allocated. In total, 234 of 271 (86.3%) participants were included in mITT and 209 of 271 participants (77.1%) in the PP population. The majority of participants were enrolled in South Africa (218/284, 76.8%). The first patient was enrolled on 27 January 2015 and last patient completed on 29 November 2017. Within the DS-TB mITT population and PP population, respectively 3/4 and 26/30 participants excluded from the analysis were lost to follow-up or withdrawn. In total, 234/271 (86.3%) participants were included in mITT and 209/271 participants (77.1%) in the PP population.

The first DSMC review (8 months after enrolment began) recommended a pause in trial enrolment following three deaths associated with hepatotoxicity on the experimental regimen arms. While these deaths raised concern about the contribution of the experimental regimen, it was noted that some of the deaths involved delays in the recognition and management of hepatotoxicity, and administration of concomitant potentially hepatotoxic medications. Patients already enrolled in the trial continued their allocated treatment without interruption. A full review of the safety data relating to the trial, and the earlier Phase 2 study,^6^ was undertaken and included external specialists in hepatotoxicity and the DSMC. No conclusive evidence was found supporting an unduly increased risk for severe drug-induced liver injury (DILI) on the experimental arms. The DSMC subsequently recommended resuming enrolment into the trial with additional safety monitoring in place. Nevertheless, the sponsor decided instead to pursue a Phase 3 clinical trial of a combination of BDQ with the Pa_200_MZ regimen that had demonstrated very promising bactericidal activity.^7^

### Primary outcome

The baseline characteristics of the participants are presented in Table 2. The primary efficacy results are presented in Table 3. In the PP analysis, 4/47 (8.5%) participants had an unfavourable outcome at 12 months on 6Pa_200_MZ arm compared to 1/53 (1.9%) on the control arm. The absolute difference in unfavourable outcomes was 6.6% (95% CI −2.2% to 15.4%). There were 11/57 (19.3%) patients with an unfavourable outcome on the 4Pa_200_MZ arm (absolute difference 17.4%, 95% CI −6.5% to 28.3) and 14/52 (26.9%) on 4Pa_100_MZ (absolute difference 25.0%, 95% CI −12.4 to 37.6). Of 11 assessable RR-TB participants receiving 6Pa_200_MZ one patient had an unfavourable outcome in the mITT analysis (withdrawn due to AE); in the PP population, none of the 10 participants had an unfavourable outcome.

**Table 2:** Baseline characteristics (mITT population)

	DS-TB					RR-TB
	
	Total* (*n* = 234) *n* (%)	HRZE (*n* = 60) *n* (%)	4Pa_100_MZ^†^ (*n* = 57) *n* (%)	4Pa_200_MZ^†^ (*n* = 61) *n* (%)	6Pa_200_MZ^†^ (*n* = 56) *n* (%)	6Pa_200_MZ^†^ (*n* = 11) *n* (%)
Male sex	167 (71.4)	42 (70.0)	46 (80.7)	42 (68.9)	37 (66.1)	5 (45.5)
Age, years, median (min–max)	34.0 (18.0–77.0)	32.5 (19.0–69.0)	37.0 (18.0–60.0)	37.0 (18.0–77.0)	31.0 (18.0–64.0)	28.0 (20.0–43.0)
Weight, kg, median (min–max)	53.0 (32.2–137.8)	54.9 (34.0–107.5)	51.3 (37.0–137.8)	53.0 (35.6–81.4)	52.6 (32.2–82.0)	55.7 (43.1–74.0)
Black	164 (70.1)	41 (68.3)	41 (71.9)	43 (70.5)	39 (69.6)	8 (72.7)
Smoking history						
Never	81 (34.6)	16 (26.7)	23 (40.4)	20 (32.8)	22 (39.3)	6 (54.5)
Past	41 (17.5)	14 (23.3)	7 (12.3)	14 (23.0)	6 (10.7)	2 (18.1)
Current	107 (45.7)	29 (48.3)	24 (42.1)	27 (44.3)	27 (48.2)	3 (27.3)
Missing	5 (2.1)	1 (1.7)	3 (5.3)	0 (0.0)	1 (1.8)	0 (0.0)
HIV-positive	58 (24.8)	15 (25.0)	13 (22.8)	13 (21.3)	17 (30.4)	6 (54.5)
Resistant to INH	0 (0.0)	0 (0.0)	0 (0.0)	0 (0.0)	0 (0.0)	5 (45.4)
CXR cavitation present	170 (72.6)	41 (68.3)	40 (70.2)	43 (70.5)	46 (82.1)	10 (90.9)
Baseline TTP ≥ median	125 (53.4)	28 (46.7)	28 (49.1)	34 (55.7)	35 (62.5)	11 (100.0)

* Includes control arm but does not include the (non-randomised) RR-TB patients.

^†^ 200 mg pretomanid (Pa, PMD) daily, 400 mg moxifloxacin (M) and 1500 mg pyrazinamide (Z) for 6 months (6Pa_200_MZ) or 4 months (4Pa_200_MZ); 100 mg pretomanid daily for 4 months in the same combination (4Pa_100_MZ).

mITT = modified intention-to-treat; DS-TB = drug-susceptible TB; RR-TB = rifampicin-resistant TB; H, INH = isoniazid; R = rifampicin; Z = pyrazinamide; E = ethambutol; M = moxifloxacin; Pa = pretomanid; CXR = chest X-ray; TTP = time to positive result (in liquid culture).

**Table 3:** Primary efficacy outcomes

	DS-TB					RR-TB
	Total*	HRZE	4Pa_100_MZ^†^	4Pa_200_MZ^†^	6Pa_200_MZ^†^	6Pa_200_MZ^†^
Modified intention-to-treat analysis						
Total randomised	271	68	65	71	67	13
Unassessable	37	8	8	10	11	2
Assessable	234	60	57	61	56	11
Unfavourable, *n* (%)	55 (23.5)	8 (13.3)	19 (33.3)	15 (24.6)	13 (23.2)	1 (9.1)
Adverse event	17	2	3	3	9	1
Withdrawal (culture-positive)^‡^	16	0	9	6	1	0
Confirmed relapse	4	0	2	1	1	0
Death (not violent or accidental)^§^	4	0	1	1	2	0
Non-adherence to study protocol	6	4	1	1	0	0
Physician decision	4	2	0	2	0	0
Death (TB-related)^¶^	2	0	1	1	0	0
Clinical deterioration in follow-up	1	0	1	0	0	0
Lost to follow-up (during treatment)	1	0	1	0	0	0
Favourable, *n* (%) (95% CI)	179 (76.5)	52 (86.7)	38 (66.7)	46 (75.4)	43 (76.8)	10 (90.9)
	(70.5 to 81.8)	(75.4 to 94.1)	(52.9 to 78.6)	(62.7 to 85.5)	(63.6 to 87.0)	(58.7 to 99.8)
Difference from HRZE in unfavourable, % (95% CI)	—	—	20.0 (−5.0 to 35.0)	11.3 (−2.6 to 25.1)	9.9 (−4.1 to 23.9)	—
Per-protocol analysis						
Total randomised	271	68	65	71	67	13
Unassessable	62	15	13	14	20	3
Assessable	209	53	52	57	47	10
Unfavourable, *n* (%)	30 (14.4)	1 (1.9)	14 (26.9)	11 (19.3)	4 (8.5)	0 (0.0)
Withdrawal (culture-positive)^‡^	16	0	9	6	1	—
Confirmed relapse	4	0	2	1	1	—
Death (not violent or accidental)^§^	4	0	1	1	2	—
Death (TB-related)^¶^	2	0	1	1	0	—
Treatment failure	2	1	0	1	0	—
Other	2	0	1	1	0	—
Favourable, *n* (%) (95% CI)	179 (85.6)	52 (98.1)	38 (73.1)	46 (80.7)	43 (91.5)	10 (100.0)
	(80.1 to 90.1)	(89.9 to 100.0)	(59.0 to 88.4)	(68.1 to 90.0)	(79.6 to 97.6)	(69.2 to 100.0)
Difference from HRZE in unfavourable, % (95% CI)	—	—	25.0 (−12.4 to 37.6)	17.4 (−6.5 to 28.3)	6.6 (−2.2 to 15.4)	—

* Total includes control arm but does not include the (non-randomised) MDR-TB patients.

† 200 mg pretomanid (Pa, PMD) daily, 400 mg moxifloxacin (M) and 1500 mg pyrazinamide (Z) for 6 months (6Pa_200_MZ) or 4 months (4Pa_200_MZ); 100 mg pretomanid daily for 4 months in the same combination (4Pa_100_MZ).

‡ Patients who failed to achieve sustained culture-negative status and were withdrawn based on clinical assessment of treatment failure.

§ During treatment.

¶ During follow-up.

DS-TB = drug-susceptible TB; RR-TB = rifampicin-resistant TB; H = isoniazid; R = rifampicin; Z = pyrazinamide; E = ethambutol; M = moxifloxacin; Pa = pretomanid; CI = confidence interval; MDR-TB = multidrug-resistant TB.

### Secondary endpoints

**Figure 2:**
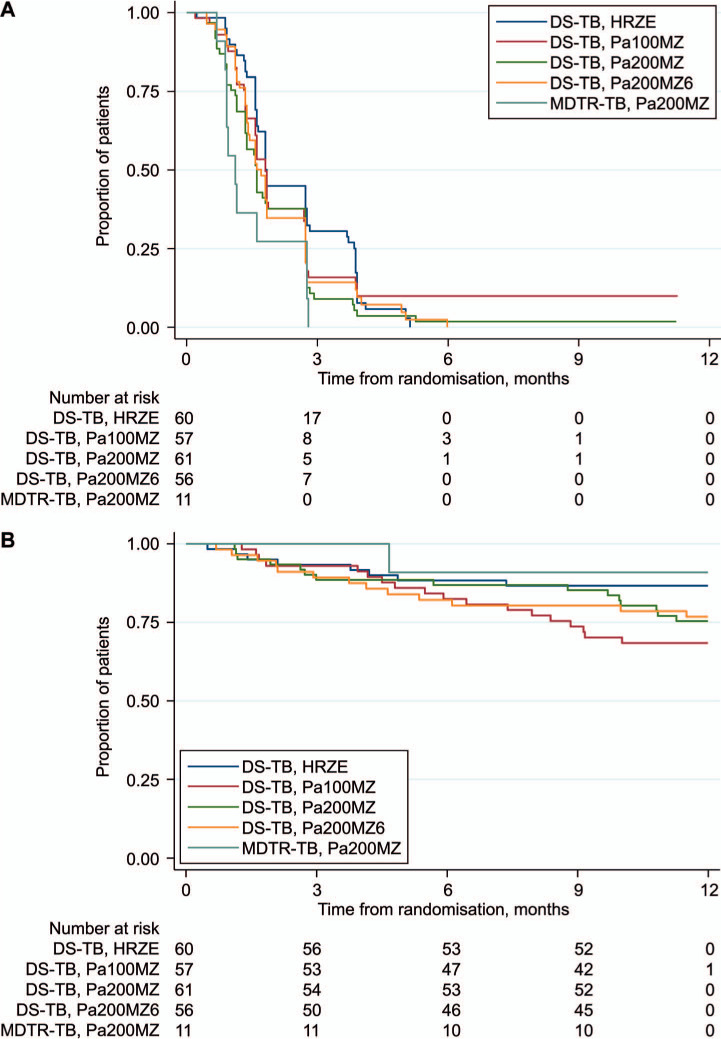
KM curves: A) time to first culture-negative status by trial treatment arm; and B) time to an unfavourable outcome by treatment arm in the modified intention-to-treat analysis.

Figure 2 contains Kaplan-Meier curves for time to culture-negative status and time to unfavourable outcome by treatment arm for DS-TB participants. There were 31/57 (54.3%) culture-negative patients at 8 weeks on control, compared to respectively 35/53 (66.0%), 36/58 (62.1%) and 33/54 (61.1%) on the 6Pa_200_MZ, 4Pa_200_MZ, and 4Pa_100_MZ arms in the mITT population (Supplementary Tables S13.9 and S13.10).

At 24 months, respectively 14/55 (25.0%), 16/57 (28.0%), 19/54 (35.0%) and 10/56 (17.9%) assessable DS-TB participants who had received 6Pa_200_MZ, 4Pa_200_MZ, 4Pa_100_MZ and control had an unfavourable outcome in the mITT population. Patients receiving 6Pa_200_MZ had 4.8% (95% CI − 6.48 to 15.97) more unfavourable outcomes than controls in the PP and 7.6% (95% CI −7.7 to 22.9) more in the mITT analysis (Supplementary Tables S13.5 and S13.6).

### Subgroup analyses

There were 14/43 (32.6%) HIV-positive participants with an unfavourable outcome on the experimental arms in the mITT analysis compared to 2/15 (13.3%) receiving the control regimen. More details can be found in Section 14 of the Supplementary Data.

### Safety analysis

In the experimental arms, 68/203 (33.5%) participants with DS-TB experienced one or more Grade 3 or higher AE (G3 + AE) while on treatment vs. 19/68 (27.9%) participants in the control arm (Table 4 and Supplementary Table S16.2). Six of 71 (8.5%) DS-TB participants on the 4Pa_200_MZ arm, 6/65 (9.2%) on the 4Pa_100_MZ arm, 11/67 (16.4%) on the 6Pa_200_MZ arm and 4/68 (5.9%) participants receiving the control regimen discontinued treatment due to an AE: most commonly related to increased liver enzymes.

**Table 4:** Safety data^ *
^

	DS-TB					RR-TB
	
	Total^†^ (*n* = 271) *n* (%)	HRZE (*n* = 68) *n* (%)	4Pa_100_MZ^‡^ (*n* = 65) *n* (%)	4Pa_200_MZ^‡^ (*n* = 71) *n* (%)	6Pa_200_MZ^‡^ (*n* = 67) *n* (%)	6Pa_200_MZ^‡^ (*n* = 13) *n* (%)
Grade 3+ AEs						
On treatment^§^	87 (32.1)	19 (27.9)	25 (38.5)	21 (29.6)	22 (32.8)	3 (23.1)
Post-treatment	21 (7.7)	3 (4.4)	4 (6.2)	11 (15.5)	3 (4.5)	0
Patients with at least one treatment-emergent AE^¶^ leading to early discontinuation from study drug	27 (10.0)	4 (5.9)	6 (9.2)	6 (8.5)	11 (16.4)	0
Top 5 treatment-emergent AEs reported for ≥5% patients by preferred term						
Hyperuricaemia	77 (28.4)	22 (32.4)	20 (30.8)	21 (29.6)	14 (20.9)	2 (15.4)
Arthralgia	73 (26.9)	15 (22.1)	15 (23.1)	24 (33.8)	19 (28.4)	4 (30.1)
Aspartate aminotransferase increased	61 (22.5)	17 (25.0)	15 (23.1)	11 (15.5)	18 (26.9)	1 (7.7)
ALT increased	53 (19.6)	11 (16.2)	13 (20.0)	11 (15.5)	18 (26.9)	1 (7.7)
Blood uric acid increased	46 (17.0)	8 (11.8)	15 (23.1)	11 (15.5)	11 (16.4)	0 (0.0)
Patients with ≥1 SAE	22 (8.1)	3 (4.4)	3 (4.6)	8 (11.3)	8 (11.9)	3 (23.1)
Liver-related SAEs	9 (3.3)	0 (0.0)	1 (1.5)	4 (5.6)	4 (6.0)	0 (0.0)
Peak ALT result						
>3xULN	40 (14.8)	5 (7.4)	9 (13.8)	12 (16.9)	14 (20.9)	1 (7.7)
>5xULN	27 (9.9)	4 (5.9)	4 (6.2)	9 (12.6)	10 (14.9)	1 (7.7)
>10xULN	16 (5.9)	2 (2.9)	4 (6.2)	5 (7.0)	5 (7.5)	1 (7.7)
Mean change from baseline in QTcF (95% CI)^#^	—	9.2 (5.4–12.9)	13.3 (10.0–16.6)	17.6 (13.8–21.5)	18.3 (15.1–21.5)	13.7 (2.5–23.8)
Deaths	12 (4.4)	2 (2.9)	4 (6.2)	3 (4.2)	3 (4.5)	1 (7.7)

* All participants who received one or more doses of study drug were included in the safety analysis.

† Includes control arm but does not include the (non-randomised) MDR-TB patients.

‡ 200 mg pretomanid (Pa, PMD) daily, 400 mg moxifloxacin (M) and 1500 mg pyrazinamide (Z) for 6 months (6Pa_200_MZ) or 4 months (4Pa_200_MZ); 100 mg pretomanid daily for 4 months in the same combination (4Pa_100_MZ).

§ Period from first dose of trial drug up to 14 days after last dose.

¶ AEs occurring between first dose of study medication and up to 14 days after last dose.

# Mean change from baseline in QTcB interval across visits for readings taken on or after the first administration of trial drug up to and including 14 days after the last administration of trial drug.

DS-TB = drug-susceptible TB; RR-TB = rifampicin-resistant TB; H = isoniazid; R = rifampicin; Z = pyrazinamide; E = ethambutol; M = moxifloxacin; Pa = pretomanid; AE = adverse event; ALT =alanine aminotransferase; SAE = serious adverse event; ULN = upper limit of normal; CI = confidence interval.

At least one liver-related treatment-emergent AE (TEAE) was reported among 61/216 (28.2%) participants with DS-TB receiving the experimental regimens: 24/67 (35.8%) on 6Pa_200_MZ, 17/71 (23.9%) on 4Pa_200_MZ, and 19/65 (29.2%) on 4Pa_100_MZ. In the control arm, 21/68 (30.9%) participants had at least one-liver related TEAE. One case met the criteria for Hy’s Law^8^ on the control arm; 23/203 (11.3%) participants receiving experimental regimens had a peak ALT of >5xULN, compared to 4/68 (5.9%) participants receiving standard TB treatment.

There were 12 deaths among participants with DS-TB and 1 death among those with RR-TB (Table 5). Two deaths on the 4Pa_200_MZ arm and one death on the 4Pa_100_MZ arm were attributed to hepatotoxicity and assessed as possibly related to the study treatment; delay in recognition of DILI and withdrawing drug were identified as contributing factors (see Section 16.2 of the Supplementary Data for more details).

**Table 5:** Deaths in the trial

Treatment group*^†^	Trial day (post-randomisation)	Trial status	Cause of death	Relationship of adverse event(s) to trial drug^‡^
DS-TB 4Pa_100_MZ	Day 482	Follow-up	Fell down from bulldozer at work	Not related
	Day 305	Follow-up	Haematemesis	Not related
	Day 39	On treatment	Fulminant liver failure	Possibly related
	Day 436	Follow-up	Massive haemoptysis	Not related
DS-TB 4Pa_200_MZ	Day 343	Follow-up	Sepsis	Not related
	Day 28	On treatment	Hepatotoxicity	Possibly related
	Day 34	On treatment	Liver failure with hepatic encephalopathy	Possibly related
DS-TB 6Pa_200_MZ	Day 163	Follow-up	Natural causes (unknown)	Unlikely related
	Day 114	On treatment	Post-mortem chest X-rays indicated pneumothorax	Not related
	Day 469	Follow-up	Poorly differentiated squamous cell carcinoma of the anal canal	Not related
DS-TB 2HRZE/4HR	Day 576	Follow-up	Left lobar pneumonia	Not related
	Day 707	Follow-up	Lower respiratory tract infection	Not related
MDR-TB 6Pa_200_MZ	Day 34	On treatment	Metastatic lung cancer	Not related

* All participants who received one or more doses of trial drug included in the analysis.

† 200 mg pretomanid (Pa, PMD) daily, 400 mg moxifloxacin (M) and 1500 mg pyrazinamide (Z) for 6 months (6Pa_200_MZ) or 4 months (4Pa_200_MZ); 100 mg pretomanid daily for 4 months in the same combination (4Pa_100_MZ).

‡ As per site investigator assessment.

DS-TB = drug-susceptible TB; H = isoniazid; R = rifampicin; Z = pyrazinamide; E = ethambutol; M = moxifloxacin; Pa = pretomanid; MDR-TB = multidrug-resistant TB.

## DISCUSSION

It was not possible to assess whether the 6Pa_200_MZ regimen was non-inferior in terms of efficacy compared to the standard TB treatment regimen for the treatment of DS-TB because the trial was stopped early after Phase 2 trial data suggested that the addition of BDQ was associated with greater efficacy. Experimental arms had higher proportions of treatment failure or relapse than the standard TB regimen; however, because of the small numbers these results are inconclusive.

In the safety analysis, a higher proportion of TEAE and serious AEs was observed in the experimental arms than in the standard TB regimen among DS-TB participants, and were most commonly hepatic in nature. Overall, 11% of DS-TB participants receiving experimental regimens and 6% of participants allocated to the standard TB regimen demonstrated a peak ALT ≥5xULN. This is higher than the proportion reported in the REMoxTB trial for the standard regimen (3% for ALT ≥5xULN, and 6% for peak ALT ≥3xULN^9^), and suggests that the inclusion criteria for this trial could have recruited patients with higher risk of hepatoxicity (for example, lower permitted haemoglobin and CD4+count at baseline).

In the Phase 2 study that preceded this trial, there was little difference observed in liver-related withdrawals between the experimental arms and standard TB treatment: eight participants were withdrawn because of elevated aminotransferases on each of the two experimental arms (100 mg and 200 mg PMD arms), and six participants were withdrawn for the same reason from the standard TB regimen.^6^ However, the numbers of patients with peak ALT/AST ≥3xULN on the experimental arms in the Phase 2 study were higher than the number of liver-related withdrawals, at respectively 10/60 (16.7%) and 14/88 (15.9%) on the 100 mg and 200 mg (DS-TB and DRTB) PMD arms, compared to 7/59 (11.8%) on standard treatment. The higher incidence of clinically significant peak ALT/AST elevations seen in the current trial compared to the Phase 2 study may be due to differences in the patient population recruited, which included more HIV-positive participants with lower CD4+ counts and a lower permitted haemoglobin at baseline. Previous work has indicated that comorbidities and less favourable baseline physiological parameters are associated with higher rates of significant hepatotoxicity with TB treatment.^10–12^ The REMoxTB study recruitment was more restrictive than STAND, and it was hoped that this would represent the wider population of TB patients and enhance the generalisability of the results.

This report highlights the need to better understand the relationship between TB treatment and hepatotoxicity.^13^ Although likely to be multifactorial, oxidative stress on hepatocytes could be a major factor.^14^ Individual responses can be hard to predict, for example some participants can tolerate the same regimen when reintroduced for unknown reasons.^15^ We need to focus on the pathological basis of TB treatment DILI to improve prediction of potentially hepatotoxic regimens.

Study limitations were predominantly related to the early halt in recruitment. Phase 2 and 3 clinical trials are not powered to make statistical analyses relating to safety, and this is especially true when halted early. Analysis of data using small patient numbers can over- or under-estimate efficacy and/or safety^16^ associated with a certain intervention, although there were approximately 60 DS-TB participants per arm. Furthermore, very few TB-HIV co-infected patients were participants in the trial.

In this Phase 3 trial, the results for the experimental regimens compared to standard TB treatment in a Phase 2 study did not appear to translate into non-inferior outcomes in either the 6-month or 4-month treatment arms. However, the study failed to reach an adequate sample size, and this limits conclusions relating to the efficacy and safety data. An ongoing evaluation of the PMD safety profile remains a priority.
